# The Extracellular Matrix in Pancreatic Cancer: Description of a Complex Network and Promising Therapeutic Options

**DOI:** 10.3390/cancers13174442

**Published:** 2021-09-03

**Authors:** Benedetta Ferrara, Cataldo Pignatelli, Mélissande Cossutta, Antonio Citro, José Courty, Lorenzo Piemonti

**Affiliations:** 1 Diabetes Research Institute (HSR-DRI), San Raffaele Scientific Institute, via Olgettina 60, 20132 Milan, Italy; ferrara.benedetta@hsr.it (B.F.); pignatelli.cataldo@hsr.it (C.P.); citro.antonio@hsr.it (A.C.); 2 INSERM U955, Immunorégulation et Biothérapie, Institut Mondor de Recherche Biomédicale (IMRB), Université Paris-Est Créteil, 94010 Créteil, France; melissande.cossutta@inserm.fr (M.C.); courty@u-pec.fr (J.C.); 3 AP-HP, Centre d’Investigation Clinique Biothérapie, Groupe Hospitalo-Universitaire Chenevier Mondor, 94010 Créteil, France

**Keywords:** extracellular matrix, stroma, stiffness, solid stress, matrix remodeling, cancer-associated fibroblasts

## Abstract

**Simple Summary:**

This review depicts the principal mechanisms involved in the process of stromal desmoplasia characterizing pancreatic ductal adenocarcinoma (PDAC). The aim of this review is to point out the role of the dense extracellular matrix in worsening PDAC responsiveness to conventional therapies. In this context, a presentation of the most promising therapeutic solutions for targeting or overcoming the matrix is provided. Even though several drug compounds revealed disappointing results in clinics, other matrix factors are now becoming the focus of studies and must be further explored to develop the optimal therapeutic strategy. Bringing novel therapeutics to PDAC patients is challenging but crucial for effectively eradicating the disease and improving patient survival.

**Abstract:**

The stroma is a relevant player in driving and supporting the progression of pancreatic ductal adenocarcinoma (PDAC), and a large body of evidence highlights its role in hindering the efficacy of current therapies. In fact, the dense extracellular matrix (ECM) characterizing this tumor acts as a natural physical barrier, impairing drug penetration. Consequently, all of the approaches combining stroma-targeting and anticancer therapy constitute an appealing option for improving drug penetration. Several strategies have been adopted in order to target the PDAC stroma, such as the depletion of ECM components and the targeting of cancer-associated fibroblasts (CAFs), which are responsible for the increased matrix deposition in cancer. Additionally, the leaky and collapsing blood vessels characterizing the tumor might be normalized, thus restoring blood perfusion and allowing drug penetration. Even though many stroma-targeting strategies have reported disappointing results in clinical trials, the ECM offers a wide range of potential therapeutic targets that are now being investigated. The dense ECM might be bypassed by implementing nanoparticle-based systems or by using mesenchymal stem cells as drug carriers. The present review aims to provide an overview of the principal mechanisms involved in the ECM remodeling and of new promising therapeutic strategies for PDAC.

## 1. Introduction

**Figure 1:**
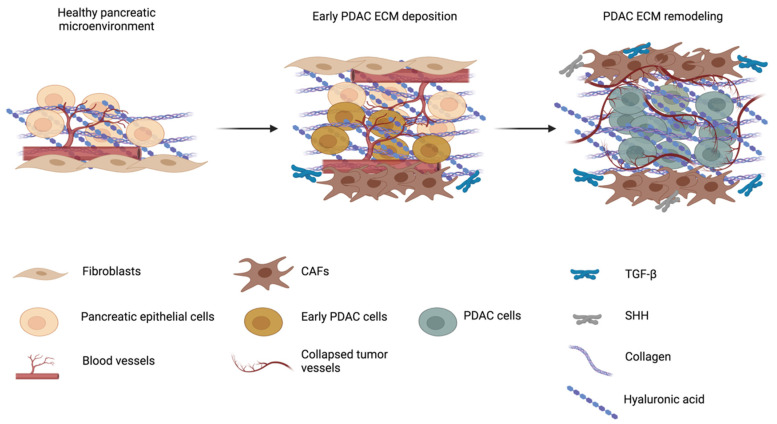
The extracellular matrix (ECM) modifications in pancreatic ductal adenocarcinoma (PDAC). In physiological conditions, pancreatic epithelial cells are surrounded by the ECM with its molecular components; cells providing structural and nutritive support, such as fibroblasts; and the vasculature network. During tumor transformation, the enhanced ECM deposition by cancer-associated fibroblasts (CAFs) is aided by molecular messengers such as the tumor growth factor ß (TGF-ß) or sonic hedgehog (SHH), and forms a dense and stiff matrix around early PDAC cells. This complex meshwork, together with the formation of new collapsed and leaky blood vessels, creates a tumor microenvironment, which favors PDAC growth and invasiveness, activating intracellular pathways that induce pro-tumorigenic programs.

Pancreatic ductal adenocarcinoma (PDAC) is a malignancy with a very dramatic clinical course and is the third largest cause of cancer-related deaths in the US, with a 5-year survival rate of lower than 10% [1]. The clinical stage of PDAC includes four classes: 1 (resectable tumor measuring between 2 and 4 cm), 2 (tumor > 4 cm, localized to the pancreas), 3 (unresectable tumor expanded to the nearby blood vessel or lymph nodes), 4 (metastatic disease) [2]. At present, the PDAC is a “silent” disease due to the absence of biomarkers and non-specific symptoms, especially in the early stages [3]. Concordantly, 80–85% of patients display a locally advanced or metastatic disease at the time of diagnosis, thereby making chemotherapy or radiotherapy the primary treatment options [4]. Even for the small subset of cases eligible for surgical resection, the prognosis remains poor and with a high risk of recurrence, especially within the first two years post-surgery [5]. In recent years, some advancements in the chemotherapeutic regimens have modestly improved the overall survival of patients. Conventional chemotherapeutic monotherapy based on gemcitabine (GCB) has been widely used in the past as PDAC standard treatment [6]. However, the therapeutic responses using GCB were disappointing. Among the tested strategies, the combination of GCB and nab-paclitaxel was reported as significantly improving the overall patients’ survival, progression-free survival and response rates [7]. Moreover, a combination of chemotherapeutic drugs (FOLFIRINOX: oxaliplatin, irinotecan, leucovorin and 5-fluorouracil) was developed and demonstrated to prolong patients’ survival when compared to GCB alone [8]. Modified (m)FOLFIRINOX was further obtained by removing the 5-fluorouracil bolus from the regimen and became the preferred adjuvant therapy for patients with PDAC who had undergone surgical resection and had not received neoadjuvant chemotherapy [9]. Despite these treatments, the drug resistance of PDAC still leads to extremely poor outcomes. The dense fibrous stroma surrounding the tumor mass, together with the abnormal vasculature network and the immune-suppressive microenvironment typical of this cancer type, are among the causes of this drug resistance [10]. The tumor microenvironment (TME) in PDAC is composed of a stiff extracellular matrix (ECM) based on collagen I, elastin and fibronectin, as well as hyaluronan (HA) and other sulfated glycosaminoglycans, which create a dense network together with surrounding fibroblasts, endothelial cells and infiltrating immune cells [11]. The remarkable ECM stiffness and desmoplasia surrounding PDAC tumor cells do not only constitute an anatomically supporting tissue, but dynamically contribute to generate a specific microenvironment facilitating tumor growth, metastasis, and survival (Figure 1) [12,13,14,15] and can constitute a barrier for chemotherapeutic drugs [16,17]. PDAC stroma is hypovascularized, presenting tortuous, compressed and poorly functional blood vessels. This phenotype is determined by different factors that can be extrinsic to blood vessels (related to the physical and chemical properties of the ECM) or intrinsic to blood vessels (related to endothelial cell activation and tumor angiogenesis) [18]. In recent years, an increasing amount of scientific evidence has highlighted the influence of the physical and mechanical properties of the tumor comprising stiffness, hypoxia and chaotic vascularization, on the drug-resistance or metastasizing abilities which are typical of this cancer [19,20,21,22,23]. Several preclinical and clinical studies have investigated numerous systems to target the ECM in PDAC. In this review, aside from describing the principal mechanisms and key players involved in the ECM remodeling, we focus our discussion on the existing or future therapeutic strategies to overcome the dense ECM of PDAC.

## 2. Cellular Component of PDAC Microenvironment: Heterogeneity and Plasticity of Cancer-Associated Fibroblasts (CAFs)

Fibroblasts are present in all solid organs, where they release several components of the matrix, cytokines and growth factors and play a role in the regulation of the homeostasis. In cancer, the activated fibroblasts have an important function in the regulation of tumor growth, dissemination and metastasis [24]. PDAC is characterized by a prominent desmoplasia where the stroma components occupy more than 70% of the total tumor volume [25]. Acellular components, mainly consisting of ECM, and cellular components, including endothelial and perivascular cells, immune cells, neurons and fibroblasts, characterize the dense desmoplastic stroma [26]. All of these components are clearly identified interacting and participating in the promotion of the growth of PDAC [27]. Among the cellular components ruling tumor growth and invasive behavior, cancer-associated fibroblasts (CAFs) are one of the most important. This cell type originates from: the activated resident fibroblasts [28]; the transdifferentiation of epithelial cells or pericytes; [29] the differentiation from mesenchymal progenitor cells located into the tumor [30]; the differentiation of adipose tissue-derived stromal cells, [31] or cancer stem cells [32,33]. Moreover, CAFs can be found within the TME or around it [34]. Several studies have shown that the differentiation of fibroblasts into CAFs is triggered by various growth factors, chemokines or inflammatory cytokines, such as FGF-2, TGF-ß, IL-6, IL-10 or PDGF, expressed by cell components of the TME [35,36,37]. CAFs can display different phenotypes and functions according to the tumor tissue in which they are located [33,38,39,40]. CAFs are characterized by a certain heterogeneity, which allows us to identify several subpopulations depending on their biological function. Two types of CAFs have been described by Tuveson’s research group, using different experimental biological models including co-cultured cells and organoids, followed by data validation in human pancreatic tumors [41]. The first type is represented by the inflammatory CAFs (iCAFs) which display a low expression of α-smooth muscle actin (α-SMA) and a high expression of inflammatory cytokines such as interleukin-6 (IL-6), IL-11 and leukemia inhibitory factor (LIF). The second type is represented by the CAFs identified as myofibroblasts (myCAFs), with a high expression of α-SMA and a low production of inflammatory cytokines. Interestingly, immunohistological studies have indicated that there is a different localization of these two CAFs types in the TME. Indeed, while myCAFs are located close to tumor cells, the majority of iCAFs are found to be distant from the tumor foci [41]. Using single-cell RNA sequencing, the existence of myCAF and iCAF is further confirmed and a gene signature is defined [42]. Using this approach, a third class of CAFs expressing MHC class II-related genes is identified [42]. This subclass of CAFs named «antigen-presenting CAFs» (apCAFs) induces T-cell receptor ligation in CD4+ T cells in an antigen-dependent manner. The existence of this heterogeneity in CAFs and their plasticity to acquire different phenotypes is confirmed by other studies [43,44]. In contrast to insights on the capacity of normal fibroblasts to inhibit cancer growth [45,46], several reports demonstrate that CAFs promote tumor growth through different pathways, including an abnormal production of ECM components, as well as matrix-remodeling molecules such as heparanase and matrix metalloproteinases (MMP) [47,48,49]. CAFs also express regulatory molecules, such as growth factors that affect tumor angiogenesis, and participate in the activation of quiescent fibroblasts [50,51,52], chemokines generating an immunosuppressive TME [53], and cytokines inducing inflammation [41,54]. All of these characteristics make CAFs a real “cellular conductor” that truly controls tumor growth in the primary tumor and metastases [55,56,57], also playing a role in the acquisition of drug resistance [58,59,60,61]. The protumorigenic potential of CAFs has prompted studies to target this cell type as a therapy for the PDAC.

## 3. Physical and Mechanical Modifications of the Matrix in PDAC

### 3.1. Fibrosis and ECM Remodeling Affect Pancreatic Microenvironment

Fibrosis is a pathological process that induces changes in ECM composition and organization, leading to scar formation within tissues during dysregulated wound repair. It brings to substitution of normal structures with fibrotic ECM, invasion and proliferation of mesenchymal cells, completely affecting tissue functions [62]. The fibrotic process is caused by the aberrant activity of the ECM remodeling machinery, affecting its composition and physical properties. Consequently, the ECM modification might induce an altered cellular response that, in the chronic wound healing processes, can culminate in the malignant proliferation and migration of cells, which is the prelude to tumorigenesis [16,63]. Fibrosis characterizes the desmoplasia of PDAC (Figure 1) [64]. Here, pancreatic tumor cells can exploit fibrotic mechanisms in order to sustain and maintain an environment suitable for their proliferation and invasiveness ability. In fact, native interstitial and basement membrane ECM are replaced by a huge amount of fibrotic ECM, which consist of collagen, especially type I, III and IV, HA, laminin and fibronectin [65]. They are mainly synthetized by PDAC cells and CAFs, which are first recruited by tumor cells upon the secretion of sonic hedgehog (SHH), TGFβ1, FGF2 and PDGF, and subsequently stimulated by immune cells, such as macrophages, attracted by the inflammatory environment [66,67]. CAFs acquire a myofibroblast phenotype expressing α-smooth muscle actin (αSMA) upon activation and show an enhanced collagen synthesis and deposition. Moreover, they are further activated by TGFβ1 autocrine signaling, which elicits a harmful self-sustaining mechanism [68]. In healthy conditions, type I and type III collagen fibrils are present and confer structural thickness and stiffness to the ECM [69]. Collagen fibril assembly is favored by crosslinks between lysine residues through a process catalyzed by extracellular enzyme lysyl-oxidases (LOX) [70]. LOX enzymes are overexpressed in PDAC, increasing the crosslinking of collagen fibers, thus stiffening the ECM [68,71]. Due to their intense crosslinking, LOX can alter cell migration and invasion, and increase resistance to treatments [72]. In fact, the use of neutralizing antibodies versus these enzymes has shown that collagen crosslinking, as well as the proliferation of metastases, was reduced, suggesting that LOXs exert important roles in tumor progression and invasiveness. Moreover, after their inhibition, vessel density increased [63,68]. In PDAC, abnormal new collagen deposition increases the density, affects the composition and organization of the fibrils, and the interstitial ECM physical properties are inevitably affected. Fibrillar collagen type I is one of the molecules most involved in desmoplasia [73]. Additionally, type IV collagen and laminin are part of the basement membrane and are similarly over synthetized, causing modifications in the architecture of the surrounding microenvironment [69,74,75,76]. Particularly, laminin proteins appear to be ubiquitously distributed within the stroma, creating a discontinuous basement membrane [77]. Among the laminin, laminin 5 (consisting of subunits α3, β3, and γ2) has been shown to mediate proliferation, apoptosis, invasion, migration and epithelial-to-mesenchymal transition in vitro [78,79] and it is negatively associated with patients’ survival [80]. It interacts with cells through focal adhesion and hemidesmosomes formed via the interaction with α3β1 integrin and α6β4 integrin. Additionally, by interacting with the overexpressed α6β1 integrin, laminin 5 induces focal adhesion kinases (FAK) and AKT phosphorylation in a time-dependent manner, increasing cell survival [80,81].

HA has been largely investigated in PDAC, since it is overexpressed in neoplastic and stromal cells [82]. HA is a polysaccharide and non-sulfated GAG component of ECM, characterized by important viscoelastic properties and involved in the water uptake of tissues [17,68,69,83,84]. Its receptor, CD44, leads to the activation of different intracellular signaling pathways, including the PI3K-AKT ERK, RhoA and RAS pathways, thereby promoting cell survival, invasion and epithelial-to-mesenchymal transition [85,86]. The deposition of a high content of collagen I and HA within the neoplastic tissue (both primary or metastatic tumor) is negatively associated with survival. Other proteins are involved in ECM remodeling and are similarly important in PDAC progression. MMPs are zinc-containing endopeptidases, which are responsible for ECM degradation during the migration and invasion of cells and, therefore, allow metastasis. More specifically, MMP-2, 7, 9 and 14 are identified as overexpressed in PDAC patients. Particularly, MMP2, secreted by activated fibroblasts, turns on the membrane-associated MMP14 (also called MT1-MMP) at the filopodia level of tumor cells, degrading the basement membrane and inducing cell extravasation. Of note, both MMP2 and MMP14 have been shown to cleave laminin 5, exposing a domain recognized by α3β1 integrin and/or α6β4 integrin and fostering cell migration and invasion [80,87]. Moreover, MMP7-deficient mice with Kras-driven PDAC showed a smaller tumor mass and less liver and lymphatic metastasization, further suggesting that they play a role in PDAC progression [88,89,90].

Aside from inducing new ECM deposition, stromal cells can also secrete diffusible factors acting on blood vessels. For instance, tumor-associated macrophages (TAMs) have been shown to produce several factors, such as the vascular endothelial growth factor-A (VEGF-A), basic fibroblast growth factor (FGF-2), urokinase-type plasminogen activator (uPA) and matrix metalloproteinase 9 (MMP9), promoting tumor angiogenesis and vascular permeability [91,92,93]. It is important to note that FGF-2 promotes endothelial cell migration in vitro, increases VEGF synthesis and induces the synthesis of collagen, fibronectin and proteoglycans by endothelial cells, reinforcing both tumor angiogenesis and the desmoplastic reaction in PDAC tumors [94,95].

### 3.2. ECM Stiffness, Solid Stress and Interstitial Fluid Pressure

**Figure 2:**
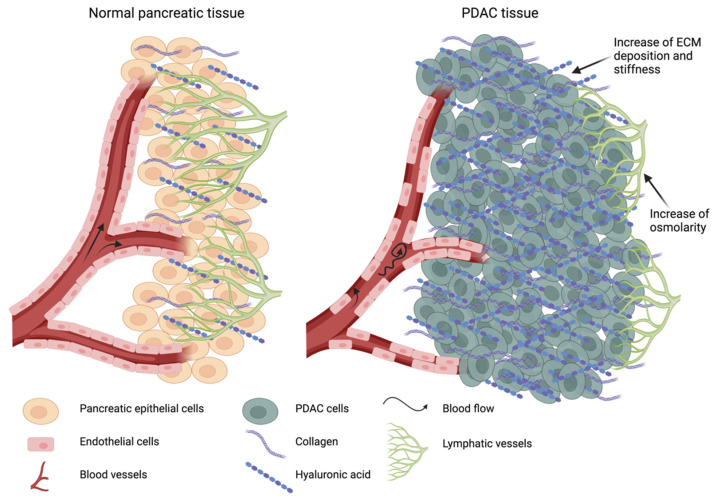
Consequences of ECM stiffness and solid stress. In normal conditions, hyaluronic acid (HA) and collagen fibers are well organized in the pancreatic tissue. There is no stress throughout the tissue and fluids can easily flow from the blood vessels to the interstitium. Functional lymphatic vessels can drain out of the interstitium, keeping its fluid pressure lower than the intravascular pressure. In PDAC, the high deposition of new ECM increases the stiffness, as well as the solid stress within the tumor mass. Meanwhile, HA increases the osmolarity of the interstitial space, inducing an augmented water uptake from the blood vessels. This, along with the fluid leakage and collapse of both blood and lymphatic vessels, increases the IFP, causing an altered fluid flow.

As mentioned above, the deposition of new ECM not only affects the microarchitecture of pancreatic tissue, but also increases the TME stiffness [63,68]. HA and collagen deposition and intense crosslinking fibrils make ECM more dense and less porous (Figure 2). Several studies investigated PDAC stiffness compared to normal pancreatic tissue, using direct rheological analysis or elastography techniques. Through analysis of the steady-state modulus, it is possible to demonstrate that PDAC biopsies are stiffer than normal pancreatic tissues, while a map based on tissue stiffness is generated through ultrasound-based elastography [96,97,98,99,100]. In vitro analysis of both collagen I and HA hydrogels showed the role of both polymers within the ECM. Collagen is predominant in stiffening matrices, as its concentration increases [101]. On the contrary, HA concentration augmentation has shown a decrease in stiffness and a shift toward fluid-like properties, but elevated resistance to compressive stress increases failure stress [102,103,104,105]. The elevated resistance to compression is due to the hydraulic resistance granted by HA [101].

Furthermore, as reported by Nia et al., the other two physical aberrancies can be identified in the desmoplastic TME: elevated solid stress and elevated interstitial fluid pressure [106,107,108]. If the stiffness is related to the ECM composition and organization, then solid stress is due to the stroma cellular components. Physical forces involved in solid stress are created by the cytoskeleton filaments involved in cell movement, migration and proliferation; the interaction of cells (tumor cells or CAFs) with ECM; and the interplaying forces between the host tissues and the tumor. Specifically, it is the combination of all of the physical forces derived from the tumor growth. So, if the tumor applies a certain force towards the host tissue, the host tissue tries to respond with similar counter forces. Moreover, although ECM stiffness can remain similar during the tumor progression, the solid stress can increase, becoming less dependent on the ECM stiffness and thus inducing the mass expansion of the tumor [107]. While in normal tissues solid stress is null, PDAC displays an elevated solid stress [19]. In particular, primary tumors display a higher solid stress than metastatic ones, while ECM stiffness results are similar [108]. Such high solid stress at primary lesions leads to the collapse of lymphatic and blood vessels when subjected to this pressure [106,107,108,109]. Previously, Kras^LSL-G12D/+^; Trp53^LSL-R172H/+^; Cre (KPC) mice bearing PDAC tumor have mostly shown constricted and collapsed vessels (up to 75%) within the tumor mass, and HA and especially collagen seem to contribute to this phenomenon [17,110,111]. Therefore, both a high stiffness and solid stress cause abnormalities in fluid flow within the tumor mass. Normally, fluid exchange between blood vessels and interstitial space is mainly ruled by intra-vasculature pressure (IVP), which is higher than the interstitial fluid pressure (IFP). Therefore, fluids tend to flow out of blood vessels mainly through the convection process, reaching the interstitial space. Here, lymphatic vessels drain the interstitium collecting the fluids. This prevents an increase of pressure within the interstitium. As previously shown, in desmoplastic tumors, HA participates in increasing IFP. Its high deposition induces a higher water uptake within the tissues, swelling the fibrotic ECM and creating a gel fluid phase that contributes to the collapse of vessels [17,112]. Oppositely, the stiff collagen matrix acts against ECM swelling, limiting the over-absorption of fluids within the tumor [109]. In addition, since there are no functional lymphatic vessels, the interstitium cannot be drained and IFP further increases. On the other hand, IVP decreases in PDAC as blood vessels lose their integrity, leading to the leakage of fluids (Figure 2) [20,108]. In this condition IFP exceeds IVP, hindering normal fluid flow [97]; therefore, convection transports become negligible, while diffusion is the dominant mechanism through which exchanges occur [19]. Likewise, small molecules, as well as macromolecules, which in normal conditions are transported through convection mechanisms, are subjected to diffusive processes, reducing their penetration within tumor mass. Furthermore, together these phenomena lead to the reverse of the pressure gradient, inducing fluids to be oozed from tumor mass towards the surrounding microenvironment. This brings tumor fluids—loaded with tumor growth factors, cytokines and cancer cells—to be spilled out to adjacent tissues [19,106,109]. Finally, the collapsing of blood vessels provokes a lack of nutrient and oxygen supply to tumor mass, resulting in an acidic and hypoxic environment promoting tumor progression [113].

### 3.3. Cellular Response to Stiffness and Solid Stress

**Figure 3:**
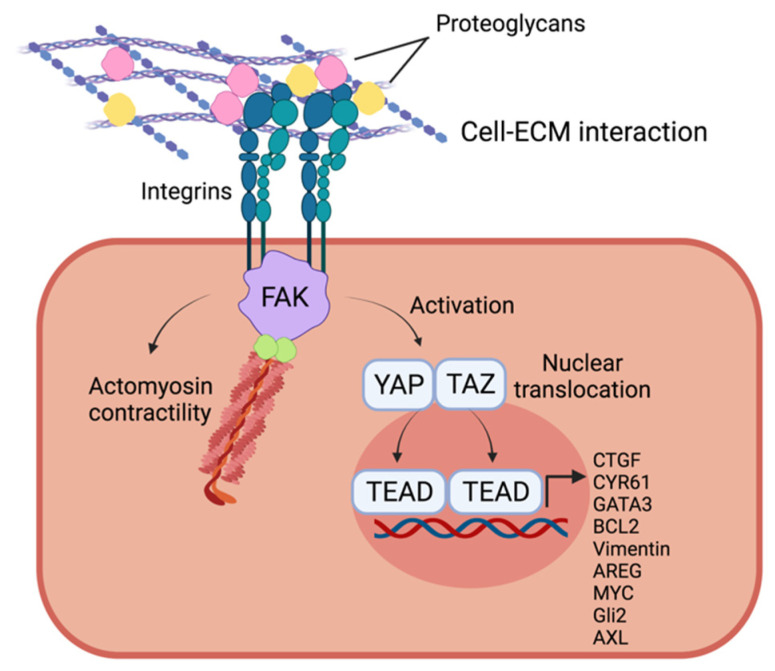
PDAC cell response to stiffness and solid stress. The interaction between ECM and PDAC cells occurs through the binding to adhesion proteins, such as integrins, expressed on the cell surface, and it is further stabilized by proteoglycans. This interaction triggers the actomyosin contractility and the activation of YAP/TAZ, which translocate to the nucleus to induce gene transcription.

Some evidence demonstrated that epithelial–mesenchymal transition (EMT), which is the prerequisite to invasion and metastasis, is also elicited by ECM stiffening. Cellular mechanosensors are involved in these processes, including proteins at focal adhesion [65]. Among adhesion proteins, integrin β1 was shown to be overexpressed in cells cultured in a rigid matrix [114]. Cells with a high number of focal adhesions with ECM have an elevated cytoskeleton tension, which is reflected by a higher phosphorylation of the actomyosin systems, responsible for cell contractility and movements [88,115]. Some heparane sulfate proteoglycans (HSPGs) intervene by stabilizing integrin β1 interaction with ECM, allowing tumor cells and CAFs to sense mechanical modifications: agrin and perlecan. Both were observed as overexpressed in PDAC, through mass spectroscopy analysis. They enhance the response to mechanical cues inducing the polymerization and reorganization of actin during cell contraction, elevating actomyosin contractility and increasing the activation of Yes-associated proteins (YAP) and their nuclear translocation (Figure 3) [90,116,117,118].

EMT is triggered by the accumulation of YAP and the transcriptional coactivator with the PDZ-binding motif, also known as WWTR1 (TAZ), within the nuclei [119,120,121,122,123]. YAP and TAZ need DNA-binding partners, as YAP and TAZ have no DNA binding ability. So, according to the partner, they can induce the expression of a wide range of genes, both tumorigenic and tumor suppressors [124]. However, when cell–cell adhesions decrease, the nuclear activated YAP/TAZ bind to TEAD, which is a transcriptional factor, accomplishing the expression of target genes: CTGF, CYR61, GATA3, BCL2, Vimentin, AREG, MYC, Gli2 and AXL. These are all genes involved in cell migration, proliferation, cell–ECM adhesion, ECM remodeling, anti-apoptotic mechanisms and cell stemness [124,125]. At this step, cells show less protein designated to maintain cell–cell interactions such as E-cadherin, causing a loss of tissue polarity, while they increase the expression of vimentin, which is a marker of mesenchymal cells [68,121]. YAP/TAZ nuclear translocation can be similarly induced by the interaction of laminin 5 to α6β4, allowing the tumor cells to maintain their stemness, as shown in epidermal stem cells [126]. Additionally, integrin β1 has been demonstrated to be involved in inducing the activation of ECM-bound TGF-β through mechanical processes. Normally, the latent TGF-β binding protein (LTBP) retains inactive TGF-β1 proteins bound to ECM. During the remodeling and stiffening of the ECM, the integrin β1 of pancreatic stellate cells (PSCs) induces the release and activation of TGF-β. This process is induced by the tension caused upon the activation of the actomyosin system [88,127]. Through mechanosensor machinery, PDAC cells have the ability to migrate and invade stiffer substrates, which is called durotaxis. Regarding this, activated PSCs have been shown to migrate according to substrate rigidity by means of integrin β1 [128]. Additionally, the in vitro analysis of different pancreatic tumor cell lines in order to evaluate cellular stiffness, has shown a growing invasiveness ability as stiffness increases [129].

Tumor solid stress can similarly induce several cell responses and can reduce cell proliferation and induce apoptosis, suggesting its role in regulating tumor morphology and growth. However, recently studies reported its role in the induction of pancreatic cell migration in vitro, by affecting the cytoskeleton organization. During PDAC development, this induces in fibroblasts an increased expression and secretion of growth differentiation factor 15 (GDF15), which is implicated in the mechanisms of tumor cell migration and invasion [107,130].

## 4. Pharmacological Tools Targeting the Stromal Barrier: From CAFs to ECM Components and Vessels Normalization

### 4.1. CAF Targeting as Therapeutic Strategy: A Double-Edged Sword

**Figure 4:**
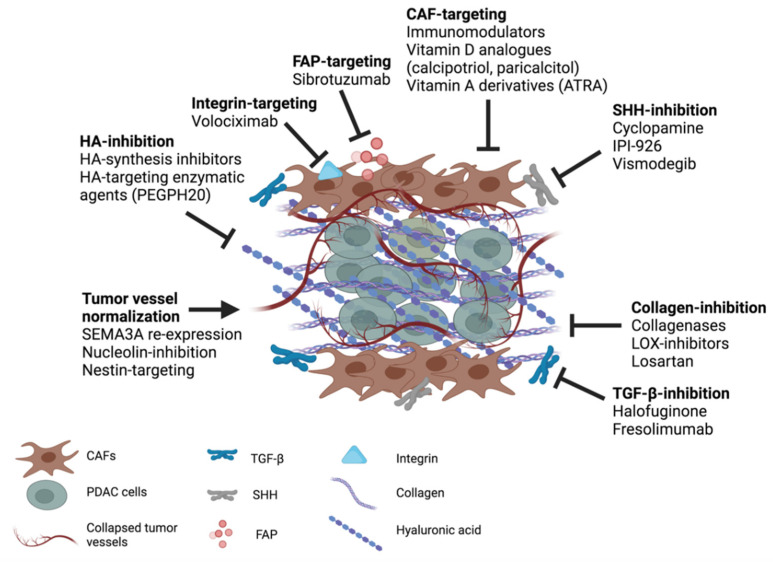
Schema of the principal drugs and pharmaceutical tools used and proposed for targeting the different components of PDAC stroma.

While many studies have focused on epithelial cells in the search for anti-PDAC therapy for several years, more recently a large number of therapeutic strategies targeting CAFs has been developed and tested (Table 1; Figure 4). The rationale of these studies comes from their tumor-promoting functions, their ability to produce tumor stromal constituents and their association with poor prognosis in cancer patients [34,131,132]. Several approaches have been undertaken, including the inhibition or the reprogramming of CAFs toward a normal phenotype. CAFs activation might be prevented by targeting the SHH signaling pathway. With this in mind, cyclopamine was proposed. It is a natural steroidal alkaloid which is able to reduce fibronectin content and to improve tumor vascularization in a PDAC xenograft mouse model. Moreover, in combination with PTX-NPs, it increases the inhibition of tumor growth by 63.3% [133,134]. In a preclinical murine model of pancreatic cancer, the administration of IPI-926, an inhibitor of SHH receptors, was combined with GCB. The treatment significantly enhanced the bioavailability of GCB in tumor tissue, inducing tumor regression. However, data obtained from clinical studies were disappointing, to the extent that the study was interrupted due to the reduced patient survival (NCT01130142) [135]. Similar results were obtained in several clinical trials involving the combination of GCB or FOLFIRINOX with vismodegib, another SHH inhibitor [136,137,138,139]. Another approach targeting CAFs expressing the fibroblast activation protein (FAP) has been investigated using diphtheria toxin. The study demonstrates that the toxin is able to enhance the anti-tumorigenic cytotoxicity of CD8^+^ T cells and to reduce tumor growth. Moreover, histological analysis of the tumor shows a reduction of CAFs migration toward the metastatic niche [14,140,141]. Analogous observations have been reported in breast and lung cancer preclinical models [14,140,142]. FAP-targeting strategies through immunotherapy have also been proposed. Sibrotuzumab, an anti-FAP antibody, was used in a phase II clinical study. The results obtained starkly contrasted with each other, with no significant effect on tumor development [143].

The role of the stroma is controversial, since it can act not only as a barrier for drug delivery but also as a protective defense mechanism that could prevent and restrain the growth of PDAC tumor. A complete stroma depletion might lead to a more aggressive cancer with a poor survival rate [26,144]. In line with these findings, other studies reported that the depletion of CAFs yielded unexpected results. In a very elegant study using transgenic approaches, mice with the ability to deplete αSMA^+^ myofibroblasts in pancreatic cancer were generated. In this model, the depletion of myofibroblasts resulted in invasive tumors with increased hypoxia and metastasis, as well as an increased infiltration of immunosuppressive cells, such as regulatory T cells, and thus decreased animal survival [145]. Similarly, targeting SHH might lead to augmented tumor progression. In a study, exploiting a well-defined mouse model of PDAC, SHH was deleted and the resultant tumors were reported to be more aggressive, presenting undifferentiated histology with an increase of tumor angiogenesis, despite the reduction of the stromal volume [146].

However, among the current therapeutic developments targeting CAFs, immunotherapy can still bring new therapeutic hopes. Indeed, strategies aimed at a vaccination against the FAP antigen in in vivo models of colon, breast and lung cancer have been proposed [147,148]. Tumor reduction is observed in lung and pancreatic cancer by the immunogenic administration of a chimeric T antigen receptor specific for FAP [149,150,151]. The targeting of CAFs either by a specific antibody or by immunotherapy remains a challenge to be accomplished by clinical validation. Many upstream studies are still required, particularly concerning the type of CAFs which need to be targeted. In fact, the heterogeneity of CAFs, which is related to their plasticity, could create a specific phenotype in different patients. Therefore, there is a necessity to address the studies of personalized medicine.

Due to the CAFs heterogeneity, several studies have been undertaken on the deactivation or reprogramming of CAFs into so-called “normal” fibroblasts [30,152]. Among the developed approaches, targeting the vitamin D receptor (VDR) through the use of analogues of vitamin D resulted in a successful reduction of fibrosis. For instance, calcipotriol reprogrammed CAFs into quiescent fibroblasts by stimulating the lipid droplets accumulation of vitamin D, which normally occurs in normal fibroblasts, and by decreasing the expression of αSMA [68,153]. In addition, the administration of calcipotriol in combination with GCB enhanced the survival of KPC mice [153]. Interestingly, a positive correlation was found between patient survival and the expression level of the VDR [154]. Currently, several trials up to phase III targeting the VDR are being investigated. For example, a randomized phase II study is being evaluated in metastatic PDAC using the combination of GCB, nab-paclitaxel and paricalcitol (NCT 03520790). Another phase Ib or II study testing paricalcitol in patients with resectable pancreatic cancer is ongoing (NCT 03300921, NCT03331562), which also uses hydroxychloroquine (NCT04524702).

Other studies have tested natural and synthetic derivatives of vitamin A, such as all-trans retinoic acid (ATRA). These molecules are strongly involved in the control of cell differentiation, growth and apoptosis. In an experimental KPC mouse model, it has been shown that ATRA administration leads to the quiescence of CAFs, causing a reduction of activated stroma, a reduction in the number of cells in the activated stroma, as well as a reduction of cancer cell proliferation [155]. An investigation of the mechanism of action indicates that this effect is mediated by the inhibition of Wnt [155]. Interestingly, it has also been shown that ATRA administration increases the infiltration of CD8-positive T cells in a KPC mouse model [156]. A randomized phase II trial testing the combination of ATRA with GCB/nab paclitaxel is currently underway (NCT04241276).

### 4.2. Targeting ECM Components

At present, several approaches have been investigated to target the dense and stiff matrix of PDAC. Collagen-targeting strategies were proposed to alleviate its excessive deposition of this tumor (Figure 4). Collagenases were proposed to degrade collagen, reducing the ECM stiffness and allowing a better delivery of drugs into the tumor site [157]. However, depending on the targeted tissues, these enzymes can have different in vivo half-lives, causing their inactivation. Therefore, some solutions were proposed for stabilizing these molecules and delivering them to the lesion site. For instance, Zinger et al. proposed collagozome, which is a 100 nm liposome encapsulating collagenase. The authors demonstrated that the treatment of the xenografts from PDAC-bearing mice with collagozome reported a strong reduction in tumor size (by 87%) when compared to mice treated with the empty liposomes and PTX [158]. However, collagen degradation may induce the release of growth factors and cytokines responsible for the initiation of the inflammatory cascade and tumor progression [159]. Therefore, the right timepoint for initiating this treatment should be cautiously defined and validated. Another approach for reducing collagen deposition might be to inhibit its synthesis, by blocking TGF-β signaling which is crucial during this process. Halofuginone is an anticoccidial which revealed the ability to reduce collagen synthesis by inhibiting TGF-β signaling in preclinical models of several solid cancers, including PDAC [160]. Fresolimumab is a monoclonal antibody targeting TGF-β and is currently being investigated in several clinical trials for cancer therapy (NCT01401062 and NCT02581787) [161]. However, a treatment targeting TGF-β should be carefully defined since it is involved in both inflammatory and tumor processes [162]. Another solution for inhibiting collagen synthesis might be the use of the anti-hypertensive drug losartan, which has contributed to the inhibition of collagen synthesis in both preclinical [163] and clinical trials (clinicaltrials.gov identifier: NCT01821729). Furthermore, the inhibition of collagen cross-linking by means of LOX inhibitors may represent a promising strategy to target ECM stiffness. Even if LOX inhibition improved the delivery of chemotherapeutic agents in mouse models of PDAC [164], it might not work for cancers with an existing mature collagen mesh.

The depletion of fibrotic ECM can occur also through the silencing of protein stabilizing mRNA of ECM components. The poly(rC)-binding protein 2 (αCP2, encoded by the PCBP2 gene) stabilizes type I collagen mRNA. Li et al. proposed a siRNA for silencing the expression of the PCBP2 gene. They evaluated the silencing effect analyzing the expression of collagen I in human PSCs and NIH 3T3 mouse fibroblasts. The treatment with human or mouse PCBP2 siRNA significantly silenced the gene with a 97% knockdown of PCBP2 mRNA expression [165].

SiRNA are further developed for silencing mucin (MUC) 20, which is a glycosylated protein aberrantly expressed in PDAC. MUC20 seems to play a role in the PDAC desmoplasia and high MUC20 expression correlates with poor survival and recurrence rate. MUC20 knockdown decreased the migration and invasion of PDAC cells induced by PSCs, suggesting that MUC20 enhances PDAC progression by modulating factors secreted by PSCs [166].

Additionally, the employment of some anti-angiotensin vasodilators has shown to deplete stromal collagen and HA in tumors, simultaneously enhancing the penetration of nanomaterials throughout the tumor stroma [111,167,168,169]. Within this context, Chen et al. proposed a sequential delivery strategy by combining GCB to nitric oxide (NO), which plays a role in ruling vascular tone and remodeling [170]. Indeed, NO was demonstrated to attenuate fibrosis through the activation of cyclic guanosine monophosphate (cGMP) and soluble guanylyl cyclase (sGC) signaling [171,172,173]. These pathways seem to interfere with the activation of TGF-β signaling, inhibiting fibroblasts activation [171]. To exploit the NO property, a system based on liposomes (Lip) loaded with S-nitroso-N-acetylpenicillamine (SNAP), which is a stable NO donor, or GCB, has been developed. As expected, the expression of the intratumoral ECM marker α-SMA and collagen I is significantly reduced after treatment with Lip-SNAP and this system reported a therapeutic efficacy in vivo.

To reduce ECM stiffness, integrins have been similarly investigated as pharmaceutical targets. At focal adhesion, they are widely expressed by cancer and stromal cells and several preclinical studies assessed that their inhibition could strongly reduce tumor progression [174]. Volociximab is a monoclonal antibody targeting integrin α5β1 and reveals a therapeutic efficacy for the treatment of pancreatic, ovarian, peritoneal and renal cancer patients during clinical trials [175,176]. Additionally, the silencing of FAK reveals interesting results. As is known, cells sense the stiffness through signals from FAK, which cooperates with integrins. The use of siRNA targeting FAK improves the sensitivity to chemotherapeutic drugs in ovarian anc colon cancer [177], thus suggesting this approach might be successful for targeting highly stiff tumors, such as PDAC.

In addition to the above-mentioned drugs, other pharmaceutical tools developed to directly target PDAC stroma include MMP inhibitors. As mentioned, MMPs play an important role in the remodeling of ECM proteins. Therefore, MMP inhibitors have been investigated in various solid tumors due to their important role in the modulation of tumor stroma, and the results were promising [178]. In particular, marimastat and BAY12-9566 were evaluated as inhibitors and administered to PDAC patients. However, the clinical outcomes were disappointing since these drugs did not report a higher anticancer effect when compared to GCB [179,180]. Moreover, the combination of anti-MMP9 antibody (αMMP9) and nab-paclitaxel was studied in preclinical models of PDAC. The addition of αMMP9 further improved the animal survival, and the metastatic burden and bloody ascites was reduced with this treatment. These findings suggested that αMMP9 might exert specific stroma-directed effects that could be easily exploited in combination with currently used cytotoxic drugs for improving PDAC clinical course [181]. Another approach of interest consisted of the design of drug carriers responding to MMP. The overexpression of the MMP-9 enzyme in the ECM of PDAC triggered the drug delivery to the tumor site. For example, an MMP-9-cleavable lipopeptide was generated and incorporated into PEGylated nanosized vesicles. The PEG groups shielded the substrate lipopeptides from hydrolysis by MMP-9. As a result, the peptide-bond cleavage led to the release of the carried content. This system resulted in the efficient delivery of GCB in vitro and in vivo [182].

### 4.3. Reducing the Interstitial Fluid Pressure in the TME

As previously mentioned, both small and large molecules reach the tumor site mainly through diffusion because the desmoplastic stroma may influence their distribution in the tumor. Similarly, drug transport can be considerably reduced [183,184]. Among the pharmaceutical tools developed with the aim of targeting the ECM components, different strategies were proposed to reduce the IFP and to bypass the ECM physical barrier (Table 1; Figure 4). A formulation encapsulating pegylated recombinant human hyaluronidase 20 (PEGPH20) was proposed to enable the degradation of HA [17,185]. Its administration to tumor-bearing KC and KPC mice could deplete HA in the stroma resulting in a decreased IFP and increased diameter of intratumor vessels. PEGPH20 in combination with GCB was used in preclinical settings showing an important reduction of tumor volume, demonstrating that the tumor perfusion of chemotherapeutic agents was enhanced by the treatment. Additionally, a clinical trial (phase Ib/II) was performed using PEGPH20 combined with mFOLFIRINOX in 138 metastatic PDAC patients [186]. Unfortunately, the median survival rate was lower in this group of patients, compared to the only mFOLFIRINOX-receiving group. However, four patients receiving the combined treatment were the only patients, among the total 138, who reported a complete response, which is an extremely rare event in PDAC.

The randomized, double-blinded phase III study HALO-301 compared PEGPH20 plus nab-paclitaxel/gemcitabine (AG) with a placebo plus AG [187]. The objective response rate was higher with PEGPH20 plus AG, but there was no improvement in the duration of the response. The safety outcomes were consistent with the established profiles of PEGPH20 and AG [7,188,189]. However, the addition of PEGPH20 did not improve the overall survival in the group of treated patients. Only 494 patients were enrolled and surely a larger study would have had a different outcome. These findings strengthen the concept that more preclinical or retrospective studies need to be performed to reduce the IFP, thus targeting the stroma remodeling. However, the concept of reducing the IFP can be promising in order to overcome the ECM physical barrier and new pharmaceutical tools still need to be explored.

### 4.4. Tumor Vessels Normalization

To date, it seems that none of the therapies based on anti-angiogenic agents used alone or in combination with other antitumor therapies significantly improve the overall survival of PDAC patients, underlying the urgent need to design new strategies to overcome vascular-induced resistance to pancreatic cancer therapy. Within this context, vascular normalization has the aim of restoring tumor vessel structure and functions to decrease the hypoxia-induced mechanisms of resistance to treatment (Figure 4). Structurally, it has been shown in several tumor models that vessel normalization (i) improves the shapes and junctions between endothelial cells (ECs), (ii) promotes the covering of vessels by perivascular cells, and (iii) restores the composition and rigidity of the basement membrane [190]. Functionally, vascular normalization has been shown (iv) to improve tumor oxygenation and therefore decrease hypoxia and angiogenesis; (v) to improve tumor perfusion, promoting chemotherapy and immunotherapy access to cancer cells; (vi) to limit the intravasation of cancer cells and metastasis; and (vii) to increase the anti-tumor immune response by promoting tumor infiltration by anti-tumor T lymphocytes and polarization of macrophages from the pro-tumor type M2 phenotype, toward the anti-tumor type M1 phenotype [190].

It has been shown that semaphorin 3A (Sema3A) is expressed in ECs during angiogenesis, where it serves as an endogenous inhibitor of angiogenesis that is present in premalignant lesions and lost during tumor progression in human uterine cervical cancer [191]. The genomic and transcriptional analyses of a wide cohort of PDAC patients identified SEMA3A gene as a transcriptional target that was downregulated by N-terminally truncated p63 (TP63ΔN) in the squamous PDAC subtype [192]. Maione et al. showed that the long-term re-expression of Sema3A induces vascular normalization in RIP-Tag2 pancreatic tumor mouse models [191]. On the contrary, some molecules are upregulated during tumor progression. Nucleolin is a glycoprotein located in the nucleus of resting cells but translocated to the cell surface and the cytoplasm of proliferative cells, such as cancer cells and activated ECs [193,194,195]. Nucleolin is a cell marker of angiogenic vessels [195] and its expression is significantly increased in PDAC patients [196]. Nucleolin targeting by a synthetic antagonist, N6L, inhibits Ang-2 secretion and participates in a program of EC loss-of-activation that increases the recruitment of perivascular cells and normalizes tumor vessels [196]. Nestin is a class VI intermediate filament protein reported to be a progenitor cell marker in various tissues. The expression level of nestin increases in various tumor cells and its expression proliferates vascular ECs [197,198,199]. The expression of nestin is exclusive to small, highly proliferative blood vessels in PDAC tissues, whereas CD34 is expressed in all-sized vessels [200]. Micro-vessel density (MVD) is often reported to correlate with prognosis in various gastrointestinal cancers [201,202]. Future studies could clarify if nestin can be a predictive and prognostic marker of MVD [203]. Furthermore, nestin targeting via small interfering RNA (siRNA) has a tumor inhibitory effect in vivo via the inhibition of tumor angiogenesis in a mouse model of pancreatic cancer [200], suggesting that nestin could be a potential therapeutic target of tumor angiogenesis. It is interesting to note that nucleolin and nestin are expressed both by tumor cells and angiogenic ECs in pancreatic tumors, suggesting that potential targeted therapies could act via different cell types of the TME.

## 5. Identifying New ECM Targets

Different strategies have been developed to identify and characterize the principal players involved in the ECM remodeling. The proteomic approach has been explored for comprehensively profiling the dynamic changes in the composition of the ECM. New proteomic protocols and pipelines were developed to selectively enrich ECM proteins based on their insolubility compared to the intracellular proteins [204,205,206]. The proteomic approach may be combined with bioinformatic pipelines to reconstitute the concept of the matrisome, which refers to both ECM and ECM-associated proteins. The matrisome project aims to generate different reference matrisomes for the organisms (MatrisomeDB; http://matrisomeproject.mit.edu/ accessed on 2 July 2021) by giving information about proteins genomically predicted to encode ECM proteins, as defined by InterPro domain-based structures which represent a classical hallmark of matrix proteins [207]. Fifty five domains were found in matrix proteins and the aim was to map the expression patterns related to organ development and disease. Based on these findings, the ECM Atlas was constituted through the compilation of proteomic data sets of ECM molecules obtained from different tissues and diseases. This platform would furnish an interesting reference to access and use information from a huge pool of data [208]. To understand how the ECM regulates disease progression, the following step regards the determination of novel ECM proteins and modulators in tissue samples from tumor progression models. Indeed, Pearce et al. defined a matrix index useful for predicting poor prognosis in high-grade serous ovarian cancer [209]. The technique of Matrix-assisted laser desorption/ionization mass spectrometry imaging (MALDI-MSI) has gained interest as it combines the specificity and sensitivity of mass spectrometry with spatial information, in order to map the distribution of molecules in the tissue [210,211]. Since cancer samples present heterogeneous histological ECM structures, the MALDI-MSI is coupled with decellularization approaches and used as a successful strategy. Indeed, this combination preserves the sample histological spatial distribution and improves the identification and mapping of ECM tissue components [212]. Indeed, a better characterization of native ECM composition, distribution and organization is important for providing a deeper understanding of its function in diseases such as cancer [213]. The decellularized ECM is assumed to be identical to the composition of native ECM and to possess native mechanical properties. The in situ decellularization of tissues (ISDoT) was developed by Mayorca-Guiliani et al. for allowing tissue decellularization without risking the collapse and destruction of the delicate ECM architecture [214]. They demonstrated that ISDoT-enriched ECM molecules made it possible to perform a proteomic analysis to register ECM changes during cancer progression. This enrichment reported a high increase in proteomic coverage when compared to non-decellularized tissues. Using ISDoT samples, it is possible to catalogue the ECM and to map the spatial distribution of its components in 3D in high resolution in the normal context vs. the tumor one. The authors provided for the first time a detailed 3D characterization of the metastatic niche in breast cancer progression and identified several ECM components not previously described. The ability to map the ECM from a spatial point of view is crucial for understanding how it influences cancer cell invasiveness, and migratory and proliferative capacity.

A further approach for investigating the principal players involved in the PDAC matrix might be to explore the signature of extracellular vesicles (EVs). As far as we know, the cellular interactions and communication occur not only through direct contact between cells such as cellular gap junctions, but also by EVs which include nano- or micro-vesicles secreted by almost every cell type both in physiological and pathological conditions [215]. EVs can carry different molecules including mRNAs, miRNAs, long non-coding RNAs, proteins, lipids and carbohydrates [216,217,218]. Since EVs can be easily obtained for the different biological fluids, the detection of the molecules that they carry makes them a potential source of biomarkers for several diseases, including cancer [215,216]. Moreover, they might deliver aberrantly expressed genes or oncogenic proteins [219]. Interestingly, EVs contain matrix-degrading enzymes such as matrix MMPs, heparanases, hyaluronidases, the ECM metalloproteinase inducer (EMMPRIN) and tissue inhibitors of MMPs (TIMPs). These MMPs presented on EV surfaces, seem to govern different proteolytic activities for the turnover of the ECM, thus contributing to matrix remodeling [220,221,222,223]. For example, the localization of MMP-9 or b-1 integrin and their shedding into EVs deriving from cancer cells participate in the localized degradation and proteolysis of ECM during cell migration, and thus metastasis [224]. Tumor-derived EVs also induced the expression of MMPs in target cells. Indeed, EV-associated heat shock protein-90 released by cancer cells could induce the expression of MMP-2 which activated plasmin, a protease inducing cancer cell invasion [225]. The presence of hyaluronidase Hya11 in EVs derived from prostate cancer induce the prostate stromal cell motility by activating FAK-mediated integrin signaling, reporting that the high Hya11 promotes the progression of this cancer [226].

As previously discussed, the potency of cancer cells to migrate and invade other tissues is largely due to the acquisition of a mesenchymal cell state. The EMT is often characterized by the secretion of MMPs, which can weaken the intercellular adhesion and reduce cell polarity with implications in metastasis [227,228,229,230,231]. Several studies evidenced that EVs are involved in the EMT [232,233,234]. Indeed, once secreted from one cell type, they can induce the EMT in the recipient cells [235]. Multiple carcinoembryonic antigen-related cell adhesion molecules (CEACAMs) and ECM proteins were identified in the EVs isolated from pancreatic duct fluid of PDAC patients, indicating a potential implication in the carcinogenesis and diagnosis of PDAC [235].

As such, the presence of proteolytic molecules or proteins implicated in the EMT among EVs may constitute one of the novel sources for identifying new possible targets which modulate the structural architecture and dynamics of ECM occurring during cancer progression.

## 6. Nanomedicine as Therapeutic Strategy: Improvement of Nanoparticle-Based Systems for by-Passing the ECM

It is well known that nanoparticles (NPs) constitute a successful platform for drug delivery since they can improve the bioavailability and solubility of carried drugs. They can specifically reach the tumor site due to the enhanced permeability retention (EPR) effect, caused by the leakiness of vessels occurring during tumor angiogenesis and the impairment of lymphatic drainage [236]. Even though the use of NPs was largely investigated in preclinical and clinical trials, the tools based on NPs vehiculating anticancer molecules provided only modest benefits in terms of survival [237]. Indeed, the abnormal TME and the heterogeneity of each tumor can negatively affect the EPR effect. The difficult tumor vasculature and the dense basement membrane may limit the vascular and interstitial transport of nanocarriers. In pancreatic cancer, where the interstitial space is thick and crosslinked collagen fibers generate a stiff matrix, the extravasation of NPs into the tumor interstitium is limited [236]. The physicochemical properties of NPs can be exploited to overcome these limitations. Indeed, PEGylated NPs which are steric small particles with a size < 50 nm are able to penetrate through stroma-rich tumors, as demonstrated on the BxPC3 pancreatic cancer cell line, better than larger NPs > 50 nm [238]. Regarding particle charge, PEGylated NPs and neutrally charged liposomes display the ability to easily diffuse in ECM hydrogel and deep penetrate into tumors, while cationic NPs remain entrapped in the hydrogel [239]. However, cationic NPs exhibit a better transvascular transport by targeting endothelial cells [240]. Other studies revealed that NPs with linear and semi-flexible shapes can diffuse and penetrate more efficiently through the interstitial matrix compared with solid spherical NPs of similar size [236,241]. The in vivo biodistribution of NPs can also be influenced by the interaction of NPs with biological fluids, causing them to acquire a surface corona of biomolecules, such as proteins or lipids [242]. For example, the FDA-approved albumin-bound form of paclitaxel, Abraxane^TM^, was generated using this approach, allowing it to acquire a prolonged circulation time [243].

As an acidic pH characterizes PDAC, smart NPs with ultrahigh pH sensitivity, which change size in the acidic TME of PDAC, were developed by Lucero-Acuna and Guzman, and with this system the penetration of encapsulated anticancer drugs was improved [244]. The pH-sensitive NPs were developed by Fan et al., who proposed a system composed of membrane-disruptive macromolecules to facilitate the penetration of drugs through the stromal barrier. This nano-formulation displayed an acid-activated cytotoxicity towards both cancer cells and fibroblasts, by disrupting the cell membrane integrity in an acid-dependent manner. Therefore, the permeabilization of the stromal barrier allowed it to target and to inhibit cancer cells. This effect was demonstrated in vitro, using 3D spheroids containing both BxPC-3 cells and fibroblasts, and in vivo on xenograft BxPC-3 tumor-bearing mice, where tumor growth was strongly inhibited without severe side effects [245].

Colby et al. also proposed a novel formulation by using an expansile unit comprising a pH-responsive group, a polymerizing methyl methacrylate group, and a hydrophilic triol-linker. The diameter of the formulation could expand up to 10 times in water and it was triggered by an acid environment, so that the NPs could release the drugs directly at the tumor site. Indeed, the efficacy with paclitaxel-loaded expansile NPs was superior to the efficacy of free paclitaxel in an in vivo model of pancreatic cancer [246].

A class of novel theranostic NPs conjugated to the insulin-like growth factor 1 (IGF1) useful for the imaging and delivery of doxorubicin (Dox) was developed for PDAC treatment. Iron oxide NPs (IONPs) were employed to target the IGF1 receptor (IGF1R) which is highly expressed in many tumor cells (including PDAC), stromal fibroblasts and macrophages [247]. Moreover, IGF1R expression increased in drug-resistant cells [248,249]. Therefore, the effect of these NPs was assessed in vivo on human pancreatic cancer patient tissue-derived xenografts (PDXs). A near-infrared (NIR) dye was conjugated to the NPs, to monitor the targeting by both non-invasive optical imaging and MRI. The nano-formulation accumulated at the tumor site, which was further confirmed by histological analysis. In line with this finding, tumor growth was significantly reduced in the animals treated with IGF1-IONP-Dox compared to control groups. These results demonstrated that IGF1-IONPs for theranostics was an effective system which overcame the tumor stromal barrier and delivered Dox directly to pancreatic cancer cells [250].

As described, several efforts have been made for improving drug delivery reducing the ECM barrier. Another strategy for employing nanocarriers regards the inhibition of ECM material production [27]. For example, metalloproteinase (MMP)-2 peptides were encapsulated in hybrid liposomes for delivering an agent downregulating ECM production—pirfenidone. In a PDAC model, this system demonstrated a reduction in the production of ECM material, thus increasing the penetration of the small molecules [251]. Additionally, collagenase was delivered by liposomes to break down the ECM in PDAC, so that the enzyme was protected and went to localize at the tumor. The pre-treatment allowed paclitaxel micelles to directly reach the tumor site and the tumors of mice which received both treatments were 87% smaller than tumors of mice which received only empty liposomes before paclitaxel micelles [158].

## 7. Towards Cell Therapy-Based Approaches: Mesenchymal Stem Cells for Drug Delivery

Another innovative system to deliver drugs into the tumor site is the system based on mesenchymal stem cells (MSCs). MSCs have been recently investigated as cellular vehicles for anticancer drugs, since they present several advantages, such as feasible isolation, availability, ex vivo expansion capacity, multipotent differentiation, immunomodulatory and non-immunogenicity properties [252]. MSCs can be isolated from many sources, including bone marrow, adipose tissue, umbilical cord tissue, placenta and amniotic fluid [253,254,255,256,257]. The observation that MSCs migrate toward inflammatory microenvironments and engraft into the tumor stroma after systemic administration suggested new therapeutic approaches for delivering anti-cancer molecules directly within the tumor. Indeed, MSCs demonstrated a migration specifically to the tumor site, because they responded to tissue damage, hypoxia and inflammation. MSCs can home to the tumor stroma, being attracted to several cytokines, growth factors and proteases of the tumor [258]. Various studies confirmed the ability of MSCs to localize at the tumor site and to be distributed among many cancer cell lines, including pancreatic cancer [259,260,261]. The high rate of migration and distribution was reported in in vitro cocultures and in vivo xenografts. Several cytokines and chemokines were found to interact with MSCs receptors [262]. A number of cytokine-receptor pairs were found to be associated with the MSCs migratory ability, including SDF-1, SCF/c-Kit, HGF/c-Met, VEGF/VEGFR and adhesion molecules such as ß1 and ß2 integrins [263,264,265]. Furthermore, even though the role of MSCs in the TME still needs to be investigated and depends on the tumor type, MSCs also reported exerting intrinsic antitumor properties. For example, in a SCID mice model of pancreatic cancer, the tumor growth rate was significantly reduced after the injection of MSCs [266].

Taken together, all these findings make MSCs a suitable candidate for a therapy targeted at the tumor site. Several therapeutic approaches based on the cell-based delivery of anti-cancer agents by MSCs have been developed. After demonstrating that Dox could be uptaken by mouse bone-marrow-derived MSCs (BM-MSCs) in a significant amount without showing evident signs of toxicity, Pessina et al. investigated whether human and mouse MSCs could be loaded with the anticancer drug paclitaxel (PTX) and exert a toxic effect towards tumor cells. Therefore, they primed the cells with a concentration of a non-toxic drug for the MSCs, which rapidly incorporated PTX and slowly released it in a time-dependent manner. BM-MSCs were able to acquire and exert a potent anti-tumor and anti-angiogenic dose-dependent effect in vitro. Furthermore, when injected in immunodeficient mouse models of melanoma, they significantly reduced tumor growth. By confocal microscopy, PTX was seen to accumulate in hMSCs-primed cells and to co-localize with Golgi apparatus and derived vesicles. Despite the mechanism in which the cells release PTX, once they reached cancer cells, they released the drug in a quantity sufficient to inhibit proliferation in vitro and in vivo. It was estimated that about 25–30% of PTX was retained by PTX-primed BM-MSCs and never released [267].

Also adipose tissue derived MSCs (AT-MSCs) reported the ability to be loaded with PTX and released the drug, inhibiting tumor cell proliferation in vitro [268]. AT-MSCs were resistant to the cytotoxic effect of PTX and released the drug to a higher extent within the first 24 h. The cell-conditioned medium (CM) collected after treatment with PTX was tested on different models of human tumors, such as osteosarcoma and prostate cancer, where cell proliferation was inhibited in vitro. In a coculture assay, AT-MSCs loaded with PTX were effective against the proliferation of a leukemia cell line. The adipose tissue represents an attractive source of stem cells since it is ubiquitous and easily obtainable without using invasive methods [269]. Additionally, clinical trials using AT-MSCs in regenerative medicine have confirmed their safety so far [270,271].

Bonomi et al. demonstrated that MSCs loaded with GCB were able to inhibit the in vitro growth of a human PDAC cell line. For the first time they showed that BM-MSCs can be loaded in vitro with GCB. A concentration of 2000 ng/mL allowed GCB to block cell division but maintained cell viability and drug accumulation. They could also inhibit the proliferation of the PDAC cell line CFPAC-1. Moreover, they showed that very high concentrations of GCB did not affect the secretome of BM-MSCs, which was interesting in terms of its potential application in regenerative medicine, since MSCs produce many factors with autocrine/paracrine functions. BM-MSCs seemed to regulate the epithelial mesenchymal transition of a tumor, initiating a cell population to maintain it, therefore a therapy based on MSCs could be integrated into the tumor mass and the drug could be delivered in situ at very high concentrations difficult to obtain by intravenous injection [272,273].

Human MSCs were primed in vitro with sorafenib (SFN), in the context of glioblastoma (GB) by Clavreul et al. After demonstrating the cytotoxicity of the released SFN in vitro, SFN-primed MSCs were administered by intranasal delivery on an orthotopic model of GB. MSCs could penetrate the brain from the nasal cavity and infiltrate the tumor with a higher accumulation after 7 days. They observed that MSCs could migrate toward large or small tumors, clinically relevant since GB is highly invasive. Even if they did not obtain an anti-proliferative effect in vivo, the treatment with SFN-MSCs consistently reduced tumor angiogenesis [274].

MSCs can be introduced into the body through local delivery into the tissue or systemically. The vascular route is often preferable since it is more feasible and less invasive, but with this methodology there are some hurdles to overcome in order to allow these cells to reach the target tissue. Firstly, they have to pass through the lungs before being distributed throughout the body. Since MSCs have a diameter of 20–50 µm, while the lung microvessels are around 10, they are often entrapped in the lungs [275,276,277]. Preclinical but also clinical evidence demonstrated that the lung entrapment occurs after iv injection of MSCs. A low engraftment level was found after the iv administration of MSCs to treat graft versus host disease (GvHD), or when co-infused with hematopoietic stem cells (HSCs) to promote HSC engraftment [278,279]. The addition of a vasodilator may solve the problem of MSC entrapment in microvessels. Moreover, since MSCs secrete mediators that exert a paracrine effect on nearby cells and tissues, they may need to be administered in very close proximity to the injury site [280]. The intrahepatic arterial injection of MSCs bypassed the lung vasculature [281]. Additionally, the intracoronary injection of MSCs resulted in a significant cell retention in the cardiac tissue [282].

Moreover, current developed techniques allow us to easily detect and track MSCs once injected in vivo. Previously, the in vivo cell tracking required a post mortem analysis of sectioned tissues, but the developments in recent years in whole body and vital in vivo imaging have allowed a higher resolution and more accurate long-term analyses. MSCs can be fluorescently labelled using fluorophores linked to a specific molecule on target cells or transduced with a bioluminescent protein reporter gene. These methods require an ex vivo cell preparation before administration but are well characterized and largely employed [283,284,285].

For many diseases, a clinical utility of MSCs has been published. Several clinical trials reported interesting results in terms of the safety and efficacy of MSCs in patients with GvHD, autism, Crohn’s disease, multiple sclerosis, systemic lupus erythematosus and type 1 diabetes. A successful use of these cells has been observed in multiple organs regarding the repair of cardiovascular, spinal and lung injuries, and bone and cartilage diseases [286,287,288,289]. Therefore, thanks to the versatility of these cells, they may constitute a promising strategy for an anticancer therapy specifically directed to the tumor site by-passing the dense ECM barrier.

## 8. Conclusions

The high ECM stiffness typical of PDAC allows the tumor to displace the host tissue and grow in an uncontrolled manner. Indeed, PDAC cells take advantage of the fibrotic mechanisms occurring in the ECM for sustaining and maintaining an optimal environment for their proliferation. Moreover, the desmoplastic stroma acts as a physical barrier impairing the delivery of anticancer molecules to the tumor site. The compression of blood vessels exerted by the stroma, together with the poor tumor perfusion, limit the access of chemotherapeutic compounds, reducing their effectiveness. In recent years, several drugs targeting the ECM components and the CAFs have been developed and most of them are currently under preclinical or clinical investigation. In fact, by targeting the stroma, the penetration of anticancer agents would be enhanced. Additionally, the improvement of NP-based systems by exploiting their physicochemical properties, such as size, charge or pH-responsiveness, increases drug delivery. Furthermore, the use of MSCs for drug delivery is interesting since it presents several advantages in terms of feasibility and intrinsic properties. Altogether, the initial results of these strategies seem to be promising, even though deeper research is required to characterize new therapeutic targets in the ECM and to improve the existing systems.

## Figures and Tables

**Table 1:** List of completed, active or recruiting clinical trials for targeting the ECM in PDAC.

Drug and Study	Target	Design	Status	Phase	PDAC Tumor Stage
PEGPH20	HA				
NCT01959139		PEGPH20 + FOLFIRINOX	Active	I/II	Metastatic
NCT03193190		PEGPH20 + atezolizumab	Active	I/II	Metastatic
NCT01839487		PEGPH20 + GCB/nab	Completed	II	Metastatic
NCT04058964		PEGPH20 + pembrolizumab	Withdrawn	II	Metastatic
NCT02921022		PEGPH20 + GCB/nab + rivaroxaban	Active	NA	Advanced
Paricalcitol	CAFs				
NCT03520790		Paricalcitol + GCB/nab	Active	I/II	Metastatic
NCT03300921		Paricalcitol + pembrolizumab	Active	I	Resectable
NCT03883919		Liposomal Paricalcitol + 5-FU/leucovorin	Active	I	Advanced progressed on GCB-based therapy
NCT03519308		Paricalcitol + nivolumab + GCB/nab	Recruiting	I	Resectable
NCT04617067		Paricalcitol + GCB/nab	Recruiting	II	Advanced
NCT04524702		Paricalcitol + Hydroxychloroquine + nab	Recruiting	II	Advanced metastatic
NCT02930902		Paricalcitol + pembrolizumab + GCB/nab	Active	I	Resectable
NCT03138720		Paricalcitol + nab + GCB + Cisplastin	Active	II	Untreated, resectable, borderline and locally advanced
NCT03415854		Paricalcitol + Cisplatin + GCB/nab (single arm)	Active	II	Metastatic
Xydroxychloro-quine	CAFs				
NCT01494155		Hydroxychloroquine + Capecitabine + Radiation (single arm)	Active	II	Resectable
NCT04132505		Hydroxychloroquine + binimetinib	Recruiting	I	KRAS mutated metastatic
NCT03825289		Hydroxychloroquine + trametinib	Recruiting	I	Stage II,III,IV unresectable and metastatic
NCT04386057		Hydroxychloroquine + LY3214996	Recruiting	II	PDAC
NCT01506973		Hydroxychloroquine + GCB/nab	Active	I/II	Advanced and metastatic
ATRA	CAFs				
NCT04241276		ATRA + GCB/nab	NA	II	PDAC
Losartan	Collagen				
NCT03563248		Losartan+nivolumab + SBRT	recruiting	II	Localized
NCT01821729		Losartan + radiation	active	II	PDAC
NCT04106856		Losartan + Rx after chemiotherapy	recruiting	I	Borderline resectable or locally advanced
Volociximab	α5β1 integrin				
NCT00401570		Volociximab + GCB	Completed	II	Metastatic
IPI-926	SHH				
NCT01130142		IPI-926 + GCB	Completed	I/II	Metastatic
NCT01383538		IPI-926 + FOLFIRINOX	Completed	I	Advanced

GCB/nab, Gemcitabine + nab-Paclitaxel; FU, Fluorouracil; SBRT, stereotactic body radiotherapy; Rx, X-radiation.
